# The IL-6 rs12700386 polymorphism is associated with an increased risk of developing osteoarthritis in the knee in the Chinese Han population: a case-control study

**DOI:** 10.1186/s12881-020-01139-2

**Published:** 2020-10-09

**Authors:** Hui Yang, Xindie Zhou, Dongmei Xu, Gang Chen

**Affiliations:** 1 Department of Orthopedics, The Affiliated Changzhou No.2 People’s Hospital of Nanjing Medical University, Changzhou, 213000 China; 2 Department of Orthopedic Surgery, The Second Affiliated Hospital of Jiaxing University, Jiaxing, 314000 China

**Keywords:** IL-6, Osteoarthritis, Case-control study

## Abstract

**Background:**

This case-control study aims to examine the association between the Interleukin-6 (IL-6) rs12700386 polymorphism and the increased risk of developing osteoarthritis (OA) in the knee in the Chinese Han population.

**Methods:**

We extracted DNA from 763 subjects (352 OA patients and 411 healthy controls). The relative expression levels of IL-6 in blood samples of patients with knee OA was determined by quantitative reverse transcription PCR (qRT-PCR) and polymerase chain reaction restriction fragment length polymorphism (PCR-RFLP) was used for genotyping the IL-6 gene polymorphism.

**Results:**

We found that the IL-6 polymorphism rs12700386 enhanced patient susceptibility to developing knee OA. Based on a subgroup analysis, the risk of developing knee OA was elevated in smokers, drinkers, and subjects ≥55 years old or with BMI ≥ 25 kg/m^2^. The combination of smoking, drinking, and having the rs12700386 genotype led to an increase in the risk of developing knee OA, indicating that an underlying interaction between gene and environment exists. The rs12700386 genotype was found to be correlated with an increase in IL-6 expression. We also found that IL-6 levels were significantly higher in the CC genotype compared to the GG genotype carriers in OA patients.

**Conclusion:**

These data suggest that the rs12700386 polymorphism in the IL-6 gene leads to an increase in the risk of knee OA in Chinese Han individuals.

## Background

Osteoarthritis (OA) is representative joint disease associated with damage to the synovial joint structure and function [1] and worldwide is estimated to affect 10% of men and 18% of women who are 60 years and older [2]. Clinically, OA most frequently affects the knee joint [3] and its occurrence is influenced by obesity, smoking, joint damage, heredity, and inflammation [4]. It is a multifactorial disease with an important genetic component [5]. In the last decade, genome-wide association studies have discovered many new genetic risk factors for OA [5].

IL-6 is a four-helix cytokine containing 184 amino acids, secreted by many cell types in response to infection, cancer, and inflammation [6]. It can effectively regulate B and T cell responses and coordinate activities of both the innate and adaptive immune systems [7]. IL-6 is an important regulator of bone homeostasis which can trigger osteoclast differentiation and bone resorption [8]. Goldring et al. found that IL-6 production underwent an up-regulation by IL-1β together with matrix metalloproteinase, and that an increase in IL-6 suppressed collagen-2 production, leading to joint damage [9], while Sakao K et al. showed that there was a positive correlation between increased IL-6 expression and OA radiographic progression [10]. We therefore propose that IL-6 is a pivotal candidate gene for OA susceptibility.

Polymorphisms in the IL-6 gene, located at 7p21–24, are associated with an increased risk of OA. Singh et al. showed that there was a significant association with IL-6 rs1800795 and rs1800796 and an increased risk of OA [11] and Lv et al found that IL-6 -634G/C was associated increased susceptibility to end-stage knee OA [12]. The IL-6 rs12700386 polymorphism, located in the promoter region of 7p22 where guanine (G) is replaced with cytosine (C) in the − 2954 position, may affect the risk of OA by activating gene expression and increasing serum IL-6 levels. The minor allele (C) frequency in the population was 0.133, published in the Ensembl database. The impact of the IL-6 rs12700386 polymorphism on OA risk has never been explored in the Chinese Han population. This paper presents a case-control study of 352 OA patients and 411 healthy controls, which aimed to investigate whether the rs12700386 polymorphism is related to knee OA risk and IL-6 gene levels in the Chinese Han population.

## Methods

### Subjects

Three hundred fifty-two OA patients and 411 healthy controls were recruited from the Affiliated Changzhou No.2 People’s Hospital of Nanjing Medical University and the Second Affiliated Hospital of Jiaxing University. All were ethnic Han Chinese who were not genetically related to each other. The Kellgren Lawrence scoring (K-L scoring) system was used to radiologically diagnose each patient, the functional Lequesne index was used to assess each subject’s functional or symptomatic status, and the Visual Analog Scale (VAS) was used to evaluate pain. There were three inclusion criteria for the OA patient group. Patients must have 1) Symptoms and/or signs of knee OA, 2) Radio-graphic abnormality with a K-L grade ≥ 2, and 3) No other form of arthritis. Demographic characteristics [gender, age, smoking, alcohol consumption, and body mass index (BMI)] and clinical characteristics of disease severity [Lequesne function index, visual analog scale (VAS), erythrocyte sedimentation rate (ESR), and C-reactive protein (CRP)] were obtained from medical records. Healthy controls were selected from patients in orthopedics clinics and general surgery in one hospital while collecting samples. The exclusion criteria for the control group were; 1) History of knee or hip replacement, 2) Application of corticosteroids or bi-immunosuppressors, 3) Parkinson’s disease, or 4) Sequelae of stroke. Table 1 lists the characteristics of all the subjects. Informed consent was obtained from all the subjects, and the study received approval from the Ethics Committee of the hospital. The study was performed according to the tenets of the Helsinki Declaration. All participating patients and institutions allowed us to access the data used in this study.

**Table 1:** The general characteristics of study participants

Variable	Cases (*n* = 352)	Controls (*n* = 411)	*P*
Age (years)	61.39 ± 10.42	61.03 ± 9.89	0.629
Sex			0.884
Male	166 (47.2%)	191 (46.5%)	
Female	186 (52.8%)	220 (53.5%)	
Smoking			0.695
Yes	111 (31.5%)	124 (30.2%)	
No	241 (68.5%)	287 (69.8%)	
Alcohol			0.762
Yes	124 (35.2%)	150 (36.5%)	
NO	228 (64.8%)	261 (63.5%)	
BMI (kg/m^2^)	24.61 ± 1.31	24.58 ± 1.49	0.814
Affected leg			
Left	215 (61.1%)		
Right	137 (38.9%)		
ESR (mm/h)	18.47 ± 9.64	–	
CRP (mg/L)	19.77 ± 14.42	–	
VAS	5.66 ± 1.59	–	
Lequesnes’ index	14.50 ± 2.06		
kellgren-Lawrence grade			
II	152 (43.2%)		
III	143 (40.6%)		
IV	57 (16.2%)		

*BMI* body mass index, *ESR* erythrocyte sedimentation rate, *CRP* C-Reactive protein, *VAS* visual Analogue Scale

### Blood sampling and genotyping

A TIANamp blood DNA kit (Tiangen Biotech, Beijing, China) was used according to manufacturer’s instructions to extract patient blood samples and isolate genomic DNA from leukocytes. IL-6 concentration in the serum of 351 OA patients and 351 matched healthy controls was determined using an enzyme-linked immunosorbent assay Kit (Boster, Wuhan, China). Extracted samples were stored at − 20 °C until further use. The IL-6 rs12700386 polymorphism was characterized by a polymerase chain reaction restriction fragment length polymorphism (PCR-RFLP) with the following sequence-specific primers: 5′-GCGACAGGCCTCTCCAG TCT-3′ (forward) and 5′-GCAGTCACACCGGCTAGGTC’ (reverse). 5% of samples were randomly selected to receive repeated assays, and we found that the accuracy rate was 100%.

### Quantitative real-time polymerase chain reaction (qRT-PCR)

Trizol (Invitrogen, Carlsbad, USA) was used to isolate total RNA from the peripheral venous blood, and the oligo primer and SuperscriptII (Invitrogen) were used to reverse-transcribe the RNA to obtain cDNA. The relative gene expression of IL-6 was quantified using the Taqman method with beta-actin used as an internal reference. The forward and reverse primers for PCR used were: 5′-GAG CTTCAGGCAGGCAGTATC-3′ (forward) and 5′-GTATAG ATT CTT TCCTTTGAG GC-3′ (reverse); (IL-6); 5′-ACCACCATG GAGAAGGCT GG-3′ (forward) and 5′-CTC AGTGTAGCCCAGGAT GC-3′(reverse)’ (β-actin). Relative expression levels were analyzed using the 2-△△CT method.

### Statistical analyses

All statistical analyses were performed using SPSS 22.0 (SPSS Inc., USA) and statistical graphs were made with GraphPad Prism 5 (GraphPad Software, La Jolla, CA). A student’s t-test or χ2 test was used to assess the differences between patients and controls in the mean and frequency distribution of clinical and demographic characteristics. Observed number of genotypes was compared with a Chi-square (χ2) analysis, with the assumption that the population was in a Hardy–Weinberg equilibrium (HWE). An independent sample t-test or a one-way ANOVA was used to test the normal distribution of continuous variables, with results shown as mean ± standard deviation. We analyzed allele and genotype distributions between patients and the controls, and conducted stratified analyses according to alcohol consumption, smoking, sex, and age of subject. The odds ratio (OR) and 95% confidence interval (CI) were calculated in a logistic regression to assess the correlation between the IL-6 gene polymorphism and risk of OA. A cross-over analysis was used to assess the effects of the interactions between environmental factors, such as smoking and/or drinking, and genetic factors on the risk of OA. *P* < 0.05 implied a significant level of association. The association between the IL-6 rs12700386 polymorphism and IL-6 serum levels was evaluated using the Mann-Whitney U test.

### Power analysis

The comprehensive statistical power of our study design was analyzed using Genetic Power Calculator 33 with a significance value of 0.05.

## Results

### Characteristics of subjects in study

Detailed information of all individuals in study is shown in Table 1. The average ages of the patient and control groups were 61.39 and 61.03 years old, respectively and mean BMIs were 24.61 and 24.58 kg/m^2^, respectively. There were no significant differences between the groups in regard to age, BMI, or sex, and the distributions of smokers and drinkers were equal group. Table 1 lists the relevant indexes, including affected leg, CRP, ESR, VAS, Lequensene’s index, and the K-L grade.

### Relationship between the IL-6 rs12700386 polymorphism and OA risk

The allele and genotype distribution of IL-6 rs12700386 polymorphism is shown in Table 2. The HWE test found that there was no obvious deviation in genotypic frequency among the controls, indicating that the subjects in this study are representative of the local population. The IL-6 rs12700386 polymorphism led to an obvious increase in the risk of OA risk in dominant, homozygous, and allelic models. A logistic regression analyses showed that the CC genotype significantly increased the risk of OA (CC vs. GG: OR and 95% CI, 2.01 (1.04, 3.89), *P* = 0.045). (Table 2). Analysis of the allelic model revealed that the C allele was associated with a higher risk of OA (C vs. G: 1.38 (1.08, 1.75), *P* = 0.010) (Table 2). This significant association was also observed in the dominant models. Stratified analyses of age, smoking, drinking status, and BMI revealed that there was a significantly higher risk of OA in drinkers, smokers, those over 55 years old, and with a BMI above 25 kg/m^2^. (Table 3). The rs12700386 polymorphism however, did not affect the risk of OA in regard to the abovementioned indexes **(**Table 4**)**.

**Table 2:** The association of genotype and allele of IL-6 rs12700386 polymorphism with osteoarthritis risk

Genotype	Genotypes and alleles	Frequencies *N*(%)		OR (95% CI)	*P*
		Cases (*n* = 351)	Controls (*n* = 409)		
rs12700386					
Co-dominant	GG	193 (55.0%)	259 (63.3%)	1.00(reference)	
Heterozygote	GC	134 (38.2%)	134 (32.8%)	1.34 (0.99,1.82)	0.063
Homozygote	CC	24 (6.8%)	16 (3.9%)	**2.01 (1.04–3.89)**	**0.045**
Dominant	GG	193 (55.0%)	259 (63.3%)	1.00(reference)	
	GC + CC	158 (45.0%)	150 (36.7%)	**1.41 (1.06–1.89)**	**0.022**
Recessive	GC + GG	327 (93.2%)	393 (96.1%)	1.00(reference)	
	CC	24 (6.8%)	16 (3.9%)	1.80 (0.94–3.45)	0.075
Allele	G	520 (74.1%)	652 (79.7%)	1.00(reference)	
	C	182 (25.9%)	166 (20.3%)	**1.38 (1.08–1.75)**	**0.010**

The genotyping was successful in 351 cases and 409 controls for rs12700386;

Bold values are statistically significant (*P* < 0.05)

**Table 3:** Stratified analyses between rs12700386 polymorphism and the risk of osteoarthritis

Variable	(case/control)						
	GG	GC	CC	GC vs. GG	CC vs. GG	CC vs. GC + GG	CC + GC vs. GG
Sex							
Male	100/131	54/50	11/8	1.42 (0.89–2.25); 0.156	1.80 (0.70–4.64); 0.238	1.62 (0.63–4.12); 0.351	1.47 (0.95–2.28); 0.094
Female	93/128	80/84	13/8	1.31 (0.87–1.97); 0.214	2.24 (0.89–5.61);0.107	1.99 (0.81–4.91); 0.176	1.39 (0.94–2.06); 0.110
Smoking							
Yes	78/103	13/12	19/9	1.29 (0.57–2.94); 0.674	**2.51 (1.09–5.78); 0.029**	**2.67 (1.15–6.18); 0.025**	**2.01 (1.08–3.76); 0.029**
No	115/156	121/122	5/7	1.35 (0.95–1.91); 0.111	0.97 (0.30–3.13); 0.602	0.84 (0.26–2.69); 0.504	1.33 (0.94–1.87); 0.116
Alcohol							
Yes	71/96	39/46	13/6	1.15 (0.68–1.94); 0.687	**2.93 (1.06–8.08); 0.049**	2.80 (1.03–7.59); 0.054	1.35 (0.83–2.21); 0.259
No	122/163	95/88	11/10	1.44 (0.99–2.09); 0.058	1.47 (0.61–3.57); 0.495	1.25 (0.89–1.76); 0.222	1.45 (1.00–2.07); 0.053
Age (years)							
< 55	30/41	48/60	8/5	1.09 (0.60–2.00); 0.878	2.19 (0.65–7.35); 0.236	2.07 (0.65–6.58); 0.254	1.18 (0.65–2.13); 0.653
≥ 55	163/218	86/74	16/11	**1.55 (1.07–2.25); 0.023**	1.95 (0.88–4.30); 0.110	1.71 (0.78–3.74); 0.235	**1.61 (1.13–2.28); 0.009**
BMI((kg/m^2^)							
< 25	96/111	62/74	9/7	1.38 (0.89–2.13);0.153	1.49 (0.53–4.14);0.605	1.51 (0.55–4.13);0.453	1.01 (0.67–1.54);0.518
≥ 25	97/148	72/60	15/9	**1.83 (1.19**–**2.81);0.007**	**2.54 (1.07**–**6.04);0.049**	2.05 (0.88–4.80);0.138	**1.92 (1.28–2.89);0.002**

Bold values are statistically significant (*P* < 0.05)

**Table 4:** Comparison of studied according to IL-6 genotypes in all osteoarthritis cases

OA(*n* = 351)									
IL-6 rs12700386		GG(*n* = 193)	GC(*n* = 134)	CC(*n* = 24)	*P*	GC + CC(*n* = 158)	*P*	GG + GC(*n* = 327)	*P*
ESR, mm/h	Mean ± SD	18.14 ± 10.61	18.82 ± 8.40	19.29 ± 8.33	0.065	18.89 *±* 8.37	0.468	18.42 ± 9.76	0.558
CRP, mg/L	Mean ± SD	20.29 ± 14.16	19.49 ± 15.06	17.96 ± 14.41	0.509	19.25 ± 14.74	0.505	19.96 ± 14.52	0.477
VAS	Mean ± SD	5.64 ± 1.51	5.75 ± 1.57	5.50 ± 2.06	0.262	5.71 ± 1.65	0.698	5.69 ± 1.54	0.671
Lequesnes’ index	Mean ± SD	14.38 ± 2.04	14.70 ± 2.12	14.46 ± 1.79	0.539	14.66 ± 2.07	0.204	14.51 ± 2.08	0.144
Affected leg	Left/right, n	117/76	81/53	16/8	0.838	97/61	0.913	198/129	0.667
KL grading	III+ IV/II, n	107/86	82/52	11/13	0.305	93/65	0.588	190/138	0.288

*SD* Standard Deviation; Bold values are statistically significant (*P* < 0.05)

**Fig. 1:**
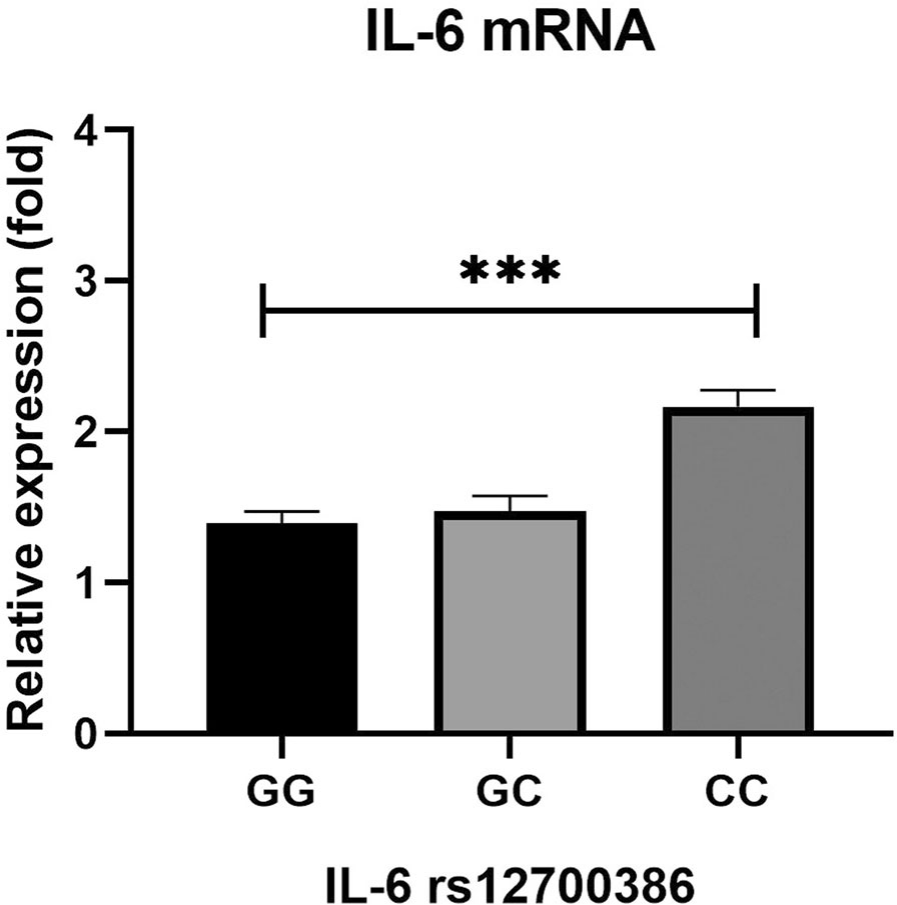
Comparison of relative expression levels of IL-6 mRNA between patients with different IL-6 rs12700386 genotypes. Analyses were performed on OA patients’ peripheral venous blood; GG (*n* = 193) vs GC (*n* = 134) vs CC (*n* = 24); Relative expressions were analyzed using the 2^△△CT^ method. The Mann-Whitney U test was used to compare the 2^△△CT^ between groups. (*** *p* < 0.001)

Based on the RT-PCR analysis, higher IL-6 expression was seen in the CC genotype compared to the GG genotype (*P* < 0.001) (Fig. 1).

### Association between IL-6 rs12700386 polymorphism and serum IL-6 levels

Data indicated that the average IL-6 serum levels were significantly higher in OA patients (P < 0.001, Supplemental Figure 1 a) and that genotype CC patients had higher IL-6 serum levels compared to genotype GG patients (*P* < 0.05, Supplemental Figure 1 b). However, this polymorphism was not associated with alteration in IL-6 serum levels in the control subjects **(**Supplemental Figure 1 c).

### Cross-over analysis

The OR and 95% CI specific to two combining exposure models (IL-6 gene variants and smoking or drinking) were calculated **(**Table 5**)**. With regards to the rs12700386 polymorphism, the GG genotype had a more obvious effect on the risk of OA than the CC genotype. While smoking was shown to have no impact on the risk of OA, smokers carrying the CC genotype were more likely to suffer from OA compared to non-smokers carrying the GG genotype (OR, 2.86, 95%CI, 1.25–6.56; *P* = 0.015). This indicated that there may be an interaction between the CC genotype and smoking. No interaction was seen between the GC genotype and smoking in terms of OA risk. A combined effect on OA risk was seen with the CC genotype of rs12700386 polymorphism and drinking, suggesting that there was an interaction between the CC genotype and drinking. There was no interaction between the GC genotype and drinking in terms of OA risk.

**Table 5:** Genetic (G) and environmental (E) factors 2*4 fork analysis

G^a^	E^b^	Case	Control	OR (95%CI); *P* value	Reflecting information
**rs12700386**					
CC vs. GG	Smoking				
+	+	19	9	**2.86 (1.25,6.56); 0.015**	G, E combined effect
+	–	5	7	0.97 (0.30,3.13); 0.602	G alone effect
-	+	78	103	1.03 (0.70,1.50); 0.923	E alone effect
-	–	115	156	1.00 (reference)	Common control
GC vs. GG	Smoking				
+	+	13	12	1.47 (0.65,3.34); 0.402	G, E combined effect
+	–	121	122	1.35 (0.95,1.91); 0.111	G alone effect
-	+	78	103	1.03 (0.70,1.50); 0.923	E alone effect
-	–	115	156	1.00 (reference)	Common control
CC vs. GG	Drinking				
+	+	13	6	**2.90 (1.07,7.83); 0.034**	G, E combined effect
+	–	11	10	1.47 (0.61,3.57); 0.495	G alone effect
-	+	71	96	0.99 (0.67,1.45); 1.000	E alone effect
-	–	122	163	1.00 (reference)	Common control
GC vs. GG	Drinking				
+	+	39	46	1.13 (0.70,1.84); 0.620	G, E combined effect
+	–	95	88	1.44 (0.99,2.09); 0.058	G alone effect
-	+	71	96	0.99 (0.67,1.45); 1.000	E alone effect
-	–	122	163	1.00 (reference)	Common control

^a^G (+): IL-6 gene rs12700386 variants (Heterozygous or homozygous); G (−): wild type

^b^E(+): smoking/non-smoking; E(−): non-smoking/non-drinking

## Discussion

In this study, we found that the IL-6 rs12700386 polymorphism led to an increased risk of OA, especially among drinkers, smokers, those older than 55 years old, and with a BMI ≥25 kg/m^2^. The interactions between the IL-6 rs12700386 polymorphism and smoking and/or drinking combined to lead to a further increase in risk for knee OA. Certain genotypes of the rs12700386 polymorphism were associated with increased IL-6 expression. We found that IL-6 levels were significantly higher in OA patients with the CC genotype compared to the GG genotype. However, the impact of rs12700386 on OA patients with regards to their clinical parameters remains unknown.

OA is characterized by the destruction of cartilage, remodeling of subchondral bone, and synovial membrane inflammation, all of which actively affect disease progression [13]. As shown in previous studies, tibiofemoral cartilage injury progression can be affected by reactive or inflammatory synovium [14]. Proinflammatory cytokines such as IL-6 and TNF, can dramatically mediate metabolic disorders as well as enhance catabolism of OA joint tissue [13]. The synovial fluid and serum of OA patients has been shown to have significantly increased IL-6 levels [15]. A clinical trial involving OA patients showed that the risk of cartilage loss increases when IL-6 and CRP have high baseline levels [16]. Increase in circulating IL-6 level and high BMI could lead to radiographic keen risk of OA [17]. In mice, IL-6 deficiency has been found to lead to a reduction in both the number of arthritic cells in the knee and the collagen-induced arthritis response [18]. However, the exact mechanism of how IL-6 affects OA is of considerable debate.

IL-6 gene polymorphisms may change gene function and expression and regulate susceptibility to osteoarthritis [11, 12]. We carried out this case-control study to explore the influence of IL-6 gene polymorphism on the risk of knee OA. Although Singh et al. found that there was no association between IL-6 rs12700386 and OA risk, they showed that IL-6 plasma levels were significantly related to specific rs12700386 genotypes [11]. However, our findings showed that rs12700386 increased susceptibility to OA. This inconsistency between studies may be attributed to 4 main factors. First, people living in different environments have different diets and lifestyles. Second, our data indicated that there is between interplay between IL-6 gene rs12700386 polymorphism and some exposure factors. Third, different genotyping approaches, as well as inclusion criteria, were used in each study. Finally, OA exhibits clinical heterogeneity which can lead to the variation of OA classification between studies.

Based on stratified analyses, smokers, drinkers, subjects older than 55, and with a BMI ≥25 kg/m^2^ had an increased risk of knee OA. The cross-over analysis was thus used to estimate the combined effects of IL-6 gene polymorphism and smoking and drinking on knee OA risk. Data indicated that smokers carrying the GG genotype and drinkers carrying the GA genotype were more prone to OA. This also suggests that gene-environment interactions could influence the occurrence of OA. The combined effects of rs12700386 polymorphisms and smoking and/or drinking enhanced susceptibility to OA. We also evaluated the associations between IL-6 gene polymorphisms and clinical characteristics of OA, however, no evidence of an association between rs12700386 and the clinical parameters of OA patients was found. To explore underlying mechanisms, qRT-PCR analysis showed that rs12700386 contributed to an increase IL-6 gene levels. We also found that IL-6 levels were significantly higher in CC genotype compared to GG genotype carriers in OA patients. Singh et al. demonstrated that genotypes of IL-6 rs12700386 was associated with increased levels of IL-6 in OA patients which was consistent with our study [11]. The possible mechanism for this may be that the change of guanine (G) to cytosine (C) at − 2954 position (−2954G/C; rs12700386) positively activate IL-6 transcriptional efficacy and hence, increases the IL-6 serum levels. Our study has shown that the IL-6 gene polymorphism can affect IL-6 gene expression and serum levels leading to an increase in the risk of knee OA.

This study does have some shortcomings. 1) only a moderate sample size was used, incapable of completely exploring how IL-6 gene rs12700386 polymorphism affected the knee OA susceptibility. 2) there may also have been some selection bias related to the ethnic groups as the participants were limited to the Chinese population. 3) only 1 polymorphism of the IL-6 gene was examined; it could not completely cover the gene. Further studies with haplotype analysis for IL-6 promoter polymorphisms are needed to profound our study. 4) relevant experiments were not carried out to fully elucidate underlying mechanisms. In further study, relevant experiments, such as transfection experiments, are need to demonstrate increased IL-6 promoter activity in the presence of the minor allele.

## Conclusions

The rs12700386 polymorphism is associated with increased levels of IL-6 gene expression and increased risk of knee OA in the Chinese Han population. These findings will be further verified in future studies utilizing a larger sample size.

## Supplementary information

**Additional file 1: Figure S1.** (a) The IL-6 serum levels of OA patients and matched heathy controls; (b) Association between IL-6 rs12700386 polymorphism and IL-6 serum levels in OA patients; (c) Association between IL-6 rs12700386 polymorphism and IL-6 serum levels in healthy controls.

## Data Availability

The datasets used and/or analyzed during the current study are available from the corresponding author on reasonable request.

## Abbreviations

IL-6: Interleukin-6
OA: Osteoarthritis
SNP: Single nucleotide polymorphism
PCR-RFLP: Polymerase chain reaction restriction fragment length polymorphism
