# Supplementary figures and images for: The IL-6 rs12700386 polymorphism is associated with an increased risk of developing osteoarthritis in the knee in the Chinese Han population: a case-control study
Source: BMC Med Genet. 2020 Oct 9;21:199. doi: 10.1186/s12881-020-01139-2 (PMC7547410; doi:10.1186/s12881-020-01139-2)

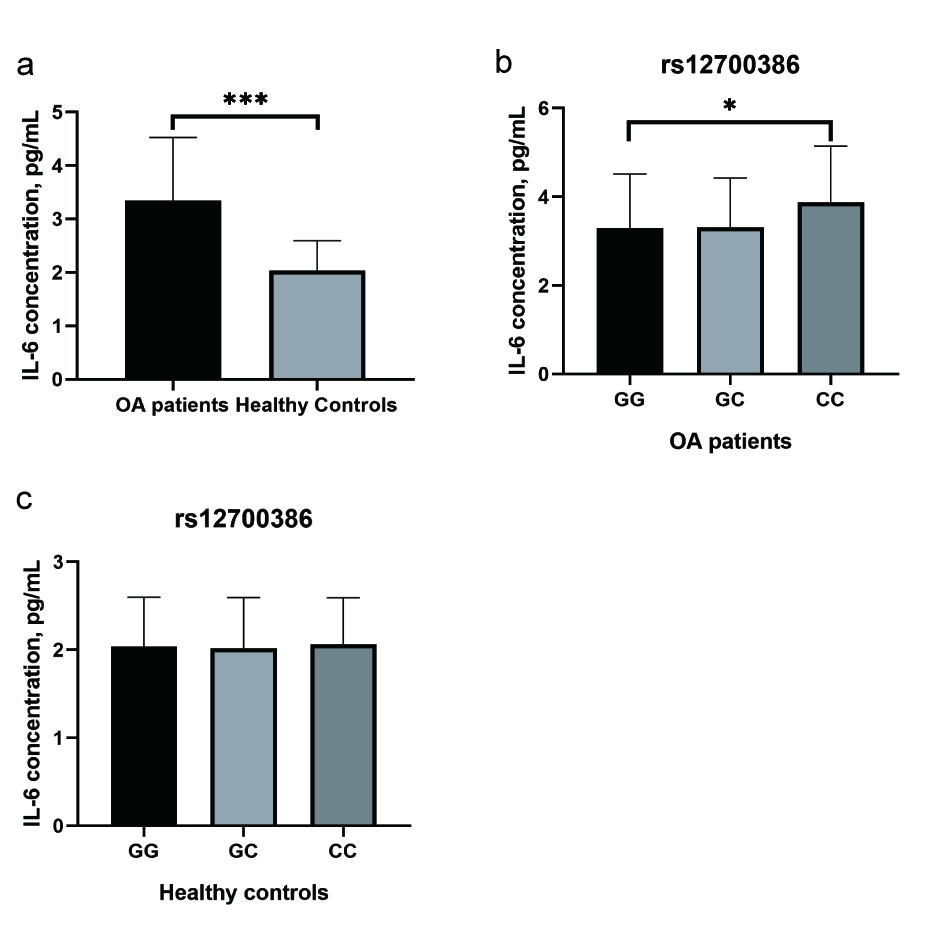

Supplement: Supplementary file 1 — Additional file 1: Figure S1. (a) The IL-6 serum levels of OA patients and matched heathy controls; (b) Association between IL-6 rs12700386 polymorphism and IL-6 serum levels in OA patients; (c) Association between IL-6 rs12700386 polymorphism and IL-6 serum levels in healthy controls. [file 12881_2020_1139_MOESM1_ESM.tif]
